# The Dual Effects of Critical Thinking Disposition on Worry

**DOI:** 10.1371/journal.pone.0079714

**Published:** 2013-11-20

**Authors:** Yoshinori Sugiura

**Affiliations:** Graduate School of Integrated Arts and Sciences, Hiroshima University, Higashi-Hiroshima City, Hiroshima Prefecture, Japan; The University of Queensland, Australia

## Abstract

This study investigated the relationship between disposition (people’s consistent motivation) toward critical thinking (CT) and worrying. In spite of its connection to psychopathology, worry is thought to represent an effort at problem-solving. Moreover, worry has been found to be underpinned by cognitive development, leading us to predict a positive relationship between worry and CT disposition. On the other hand, cognitive behavioral therapy, which involves techniques similar to CT, has been shown to be effective in reducing worrying, suggesting that increasing CT disposition decreases worrying. This study attempted to reconcile these seemingly contrasting predictions about the relationship between CT disposition and worrying by using multiple mediator analysis. A model was proposed wherein the mediators, responsibility to continue thinking and detached awareness of negative thinking, were related to two opposing predictions. The former is thought to lead to enhanced worrying and the latter to reduced worrying, with both positively related to CT disposition. A questionnaire study with university students (*N* = 760) revealed that CT disposition enhanced worrying by obliging people to continue thinking about a problem, but that it also reduced worrying by enhancing the detached and objective awareness of their negative thoughts. This study thus demonstrated the dual effects of CT disposition on worrying through different mediators. Thus, when enhancing CT disposition, it is important for educators to be aware of possible disadvantages apart from its worry-reducing effect. Future studies should therefore examine the underlying mechanisms of these two effects of CT disposition.

## Introduction

Critical thinking (CT) is defined as purposeful, reasoned, and goal-directed thinking [1]. During CT, people deliberately monitor their thinking processes and are aware of possible flaws in their reasoning (e.g., biases and premature conclusions). According to the American Philosophical Association’s Delphi Report, CT is a “liberating force in education and a powerful resource in one’s personal and civic life” [2].

In order to promote CT, it is important to understand its motivational aspects. It is widely acknowledged that CT is composed of skills and dispositions [3]. “CT disposition” refers to people’s consistent motivation to use CT in problem-solving [3], their willingness and confidence in its use, and the degree to which they enjoy it. The Delphi Research Project involved extensive discussions among experts to identify the components of CT skills and dispositions [2]. The Delphi Project stated that the disposition of an individual inclined to use CT has the following characteristics: 1) inquisitiveness with regard to a wide range of issues; 2) open-mindedness regarding divergent world views; 3) systematic in inquiry; 4) an analytical nature; 5) eagerness and courage to seek knowledge; 6) confidence in one’s ability to reason; and 7) the maturity to admit the existence of ill-structured or multi-faceted problems [4]. The importance of disposition is further exemplified by a study that found that although many medical educators describe CT as an ability, these people’s descriptions of CT in concrete scenarios actually reflected it as a dispositional factor (e.g., lack of cognitive effort) [5].

Evidence is accumulating in support of a relationship between CT disposition and the successful implementation of CT in experimental tasks. These studies typically predict performance in reasoning tasks by cognitive abilities (e.g., IQ) and cognitive styles that overlap with CT disposition. Chan, Ho, and Ku found that beliefs in certainty of knowledge predicted unsuccessful CT (e.g., appreciation of a counter-argument) beyond cognitive ability [6]. Macpherson and Stanovich also found that both cognitive ability and CT disposition (e.g., actively open-minded thinking and need for cognition) predicted successful CT (less-biased thinking) [7]. Kokis et al. found that CT disposition predicted performance in reasoning tasks beyond cognitive abilities in children aged 10–13 [8]. In addition, previous research has indicated that CT disposition has many other positive effects. For example, a self-report measurement of CT disposition (the California Critical Thinking Disposition Inventory; CCTDI) [3] predicted better grade point averages (GPAs) in students [9]. CT disposition scores were also correlated with lower library anxiety [10], lower general anxiety, higher self-esteem [11], and flexible adaptability [4].

These findings indicate that CT disposition is related not only to the use of CT and academic success, but also to indices of mental health. Therefore, it is important to investigate the relationship between CT disposition and clinically relevant variables in order to not only maximize its benefits in clinical settings, but also motivate the use of CT.

Worry is particularly important in this regard, not simply because it is a representative indicator of psychopathology [12], but because of the possible competing predictions about its relationship to CT disposition. Anxiety includes cognitive (e.g., worry), physiological (e.g., hyperarousal) and behavioral (e.g., avoidance) components [13]. The chain of negative and uncontrollable thoughts that constitutes worrying [14] is one of the cognitive components of anxiety. Although worry is a part of anxiety, studies have found that the former enhanced the latter [12]. Prolonged worrying maintains affective and physiological arousal in response to stress, and eventually leads to reduced somatic health [15]. It is also related to various anxiety disorders, indicating its clinical importance [12].

Borkovec et al. stated that worry “represents an attempt to engage in mental problem-solving on an issue whose outcome is uncertain but contains the possibility of one or more negative outcomes” (p. 10) [14]. The definition of worrying that considers it an attempt at problem-solving implies that CT disposition and worrying are closely related, because both CT and problem-solving are members of the closely related family of higher order cognitions, together with decision making and creative thinking [2]. Consistent with Borkovec’s notion that worry is related to problem-solving, researchers have found correlations between daily problem-solving and worry [16], [17]. Worry uses working memory resources [18], consistent with the notion that it is one of the higher-order cognitive skills. In addition, cognitive development (e.g., an understanding of multiple possibilities) was found to mediate age-related increase in worry elaboration in children aged 3–7 [19]. Taken together, these findings suggest a positive association between CT and worrying. Investigating possible negative effects of CT disposition is important because it may reveal clues as to why people do not engage in CT.

How can the positive link between worry and problem-solving be explained? An etiological model of worry suggests that it begins as an active attempt at problem-solving but becomes rigid and inflexible [20], [21]. A series of studies that used the Responsibility to Continue Thinking (RESP) Scale to measure rigidity and inflexibility supports this assertion [17], [21]. RESP scores reflect the belief that one is required to participate in prolonged and persistent thinking about stressful problems (e.g., “I should continue thinking until I find a better solution” and “It is irresponsible to stop thinking”). Although worry is associated with many predictors [22], RESP score is one of the strongest [21]. RESP score and worrying were found to be related to the general tendency to actively engage in problem-solving and not confined to specific aspects of problem-solving (e.g., information-seeking or solution generation) [17]. RESP score was also found to mediate the relationship between self-reported active problem-solving and worrying [17], [23]. Therefore, it is possible that CT is not exceptional among problem-solving approaches, and should be positively correlated with RESP score (and, in turn, with worrying). In other words, motivation and willingness to engage in CT as represented by CT disposition can sometimes lead to persistent thinking.

However, another line of research suggests the opposite possibility: that CT disposition is negatively related to worrying, consistent with the demonstrated relationship between CT disposition and reduced anxiety [10], [11]. Although anxiety and worry are not entirely the same [13], as no previous study has directly dealt with the relationship between critical thinking and worrying, it is informative to examine the literature on the relationship between CT disposition and anxiety for further insight. An explanation for the anxiety-reducing effect of CT comes from cognitive behavioral therapy (CBT). CBT is currently the most effective psychological intervention for various psychological disorders [24], including worrying [25], and assumes that individuals with anxiety and depression tend to think negatively in various situations. The goal of CBT is to modify such thinking through cognitive modification skills, similar to CT (e.g., objective awareness of one’s negative thinking pattern, analysis of evidence for and against one’s conceptualization of a situation, and testing the effect of an alternative way of thinking) [26].

The mechanism underlying CBT is thought to be similar to that of CT. According to CT theory, exclusive reliance on one’s habitual way of thinking (e.g., heuristics) can lead to errors; thus, one would seek to enhance awareness of possible flaws in one’s thinking [27]. The same is true for CBT. It has been found that some people experience relatively automatic (frequent, unintentional, difficult-to-control) negative thoughts about themselves [28], and the habitual nature of such thoughts was predictive of low self-esteem and anxiety, independent of the negativity of the contents [29]. However, CBT does not introduce Pollyanna-like thinking; rather, it promotes a detached awareness of negative thinking [30]. Teasdale et al. found that CBT works by enhancing detached awareness of one’s negative thinking (i.e., noticing that negative thoughts are not facts, but are merely mental phenomena) [31]. A self-report measure of detached awareness was found to be negatively related to worry [32]. Therefore, detached awareness is considered a potential mediator of the effects of CT on reduced worry.

Indeed, when previous studies found that responsibility to continue thinking mediated the positive relationship of problem-solving to worry, negative relationships between problem-solving and worrying also emerged, after controlling for the responsibility to continue thinking [17], [23]. Such negative relationships were not evident in simple correlations. In these studies, confidence in problem-solving mediated this negative relationship [17], [23]; in the present study, detached awareness is introduced as a potential mediator.

Therefore, problem-solving can both increase and decrease worrying. Consequently, CT disposition is expected to have a double-sided effect on worrying, indicating the need for a model that differentiates the positive and negative effects of CT. Based on the above literature review, it was therefore hypothesized that CT disposition can both enhance and reduce worrying, with each effect mediated by different mediators. To summarize, constructs include the following: 1) CT disposition, 2) responsibility to continue thinking, 3) detached awareness, and 4) worrying. CT disposition is expected to be positively related to both responsibility to continue thinking and detached awareness. Each relationship is derived from studies that related problem-solving with worry and responsibility to continue thinking [17], [23], or studies of the mechanisms of CBT [30], [31]. Further, as previous studies have found, the responsibility to continue thinking will enhance worry [17], [21], but detached awareness will reduce it [32]. It may seem unusual to predict a positive relationship between CT disposition and worrying when a negative relationship has already been demonstrated between CT disposition and anxiety [10], [11]. However, Paulhus, Robins, Trzesniewski, and Tracy argued that even when a simple correlation indicates the contrary, one might continue to build a hypothesis that includes opposing mediating effects [33]. Paulhus et al. based their discussion on the demonstration of suppressor effects. Suppressor effects include situations where a certain predictor behaves in a manner inconsistent with its simple regression weight (or simple correlation) to the target variable, for example, a situation when a predictor with a simple positive correlation with the target contributes to the prediction together with other correlated predictors, resulting in a negative regression weight.

The following results successfully indicated two pathways between CT disposition and worrying. In addition, a few supplementary analyses were also conducted: gender invariance of the mediational model, the effects of CT disposition subscales on worrying, and the possibility of an alternative mediational model.

## Methods

### Participants

Japanese college students (*N* = 760) in multiple introductory psychology classes (360 men and 400 women) with a mean age of 19.06 years (*SD* = 2.91) were recruited and completed a questionnaire packet. Participants completed a paper-and-pencil questionnaire in groups in the classroom. As these classes were introductory, many participants were first- (80%) or second-year students (16%). Accordingly, 44% were age 18, 37% were 19, 11% were 20; the remaining students were age 21–63. Data are available from the author upon request.

### Ethics Statement

This study was approved by the institutional ethical review board at Hiroshima University Graduate School of Integrated Arts and Sciences (20-2). Before beginning, participants were explained the nature and purpose of the study and were told that they were free to refuse to participate in the research, at which point their data would be discarded. Participants were asked to return filled questionnaires only if they agree to take part in the study. Therefore, the act of filling and returning questionnaires was considered as consent. This procedure was adopted because the questionnaires were analyzed anonymously. The research protocol submitted to the IRB stated these anonymity procedures and was approved without the explicit use of written consent.

### Materials

#### Critical Thinking Disposition (CTD) scale [34]

The CTD consists of 33 items that measure motivation for CT. Items are rated on a 5-point scale ranging from 1 (*not true*) to 5 (*true*). The CTD was developed by using a pool of items containing multiple self-report measures related to the disposition to use CT (a total of 96 items). Factor analysis with responses from 426 Japanese college students identified four factors contributing to the use of CT, namely, Awareness of logical thinking, Inquiry-mindedness, Objectiveness, and Evidence-based judgment (Appendix S1). The hierarchical factor structure with the second-order factor of CT disposition over four sub-factors was supported by confirmatory factor analysis. Therefore, the composite score of the four subscales can be used as an overall index of CT disposition. As the CTD was derived from a substantial pool of items, it can be assumed that it encompasses sufficient content areas. In particular, comparing the item contents of the CTD with the CCTDI [3], the first published and most widely used scale of CT disposition, reveals that the CTD is a valid measure. Although the CCTDI is the most widely used measure of CT disposition, there has been some concern over its validity in a Japanese population. Facione et al. suggested there may be an increased risk of response bias based on social desirability in Japan [3]. There is published information on the validity of the CTD in Japan, however; thus, it was deemed to be the most appropriate measure for this study. The CTD has been correlated with other self-report measures (the need for cognition, openness to experience, and empathy), thereby demonstrating its construct validity. Furthermore, the Inquiry-mindedness subscale predicted the use of CT during a critical reading task (accepting evidence that counters one’s hypothesis) among college students (*N* = 85) [34].

#### Responsibility to continue thinking scale (RESP) [17], [21]

The RESP Scale was developed to measure the metacognitive appraisals (how people evaluate and control their own cognitions) that occur during problem-solving in stressful situations. Participants were asked to rate how often they experience the conditions described in each item on a 5-point scale ranging from 1 (*none*) to 5 (*very frequent*). The RESP contains 14 items, reflecting the extent to which one believes that prolonged and persistent thinking about stressful problems is necessary. Adequate reliability (internal consistency coefficients αs >0.88) and validity (e.g., correlations with perfectionism and active problem-solving traits) have been reported for this scale [17]. It has been shown that the RESP explains additional variance in the degree of worrying beyond other known predictors [21], indicating its incremental validity.

#### Refraining from Catastrophic Thinking Scale (REF) [35], [36]

Detached awareness is measured via the Refraining from Catastrophic Thinking (REF) Scale. The REF is one of two factorial-derived subscales of the Cognitive Control Scale (CCS) [36]. The CCS is a face-valid measure consisting of items derived from CBT techniques [26]. Participants were asked to rate the extent to which they believed they could perform the actions described in each item when they were anxious. The REF consists of five items, with responses on a 4-point scale ranging from 1 (*I absolutely cannot*) to 4 (*I definitely can*), that measure the detached awareness of negative thinking. Specifically, it measures the ability to conceptualize thoughts as only mental events (and not reality), and the ability to suspend further worrying (e.g., “Even if the bad consequences of a problem come to mind, I can reassure myself that they are nothing more than my imagination”; “When I start thinking about the situation seriously, I can stop for a while”). A series of studies have established the reliability and validity of the REF [35], [36], and it has acceptable internal consistency (α >0.71). Furthermore, the REF was negatively correlated to indices of negative emotions both cross-sectionally and longitudinally [32], [37]. Finally, REF scores were found to have improved after a meditation-based program for outpatients with diverse mental disorders [38] and nonclinical samples [39], [40]. In addition, Katsukura et al. found that increased REF scores were related to symptom reduction, suggesting that the REF may be a mediator of therapeutic change in such interventions [38].

The other subscale, Logical Analysis, reflects active and objective problem-solving skills (e.g., “I can think of several alternatives for how to think or act”). Its contents are, therefore, quite different from the REF and do not represent detached awareness. In fact, the discriminant validity of the two CCS scales has been indicated by confirmatory factor analysis [36] and by differential relationships to other variables (e.g., logical analysis was related to active-problem-solving, while refraining from catastrophic thinking was related to reduced emotional distress) [35]. Therefore, this subscale was not included in the present study.

#### Penn State Worry Questionnaire (PSWQ) [41]

The PSWQ is a 16-item questionnaire with excellent psychometric properties that measures the frequency and intensity of worry [42]. Items are rated on a 5-point scale ranging from 1 (*not at all true*) to 5 (*very true*). The reliability and validity of the Japanese version of the PSWQ [43] are comparable with the original version. The reliability obtained in this study was also excellent (α = 0.92). Construct validity was demonstrated by a single factor structure and positive correlations with anxiety (*r* = .72; *p*<.001; *n* = 254) and depression (*r* = .51; *p*<.001; *n* = 227) in the student sample. In addition, this scale demonstrated good discrimination from obsessive symptoms (different factors were formed).

The PSWQ has been associated with cognitive, behavioral, and physiological characteristics of worrying. (1) High scorers emitted more steps in a catastrophizing task in which participants are asked to list their concerns to a consecutive chain of questions, “What is it that worries you about X?” where X is a worry topic or an answer to a preceding question. They felt more responsible for fully considering the problems than low scorers [44]. (2) High scorers experienced greater anxiety, interfering thoughts, and reduced performance in a cognitive task after false failure feedback than low scorers [45]. (3) When both the PSWQ and dispositional anxiety were high, people had difficulty in disengaging attention from angry faces [46]. (4) High scorers showed slow heart rate (HR) recovery after a stressful (unsolvable) cognitive task [47]. Similarly, high scoring women exhibited higher HRs during various tasks [48]. Taken together, those who highly endorse the PSWQ items indicate characteristic cognitive, behavioral, and physiological responses.

### Data Analysis

Descriptive statistics, alpha reliabilities, and correlations were computed by SPSS 21.0 (SPSS Inc., Chicago, IL). Mediation by responsibility to continue thinking and detached awareness was examined by multiple mediation analysis. Based on their simulation studies, Mallinckrodt, Abraham, Wei, and Russell recommend bootstrap estimation of indirect effects as a desirable way to test mediation [49]. The indirect effect is the product of path coefficients before and after the mediators. Preacher and Hayes designed a detailed procedure for bootstrap estimation of the statistical significance of the indirect effect [50], while Hayes provides an SPSS macro called “PROCESS” for this purpose [51].

Invariance of the model between genders was examined via multiple population analyses by structural equation modeling carried out with AMOS 20.0. This procedure compares fit indices between a model that specifies that its parameters are the same across genders and the other with no such specification. If the former indicated better fit, it can be concluded that model does not differ across genders.

Statistical significance was set at *p*<.05 (two-tailed).

## Results

### Preliminary Analyses

Table 1 depicts the descriptive statistics and internal consistency coefficients of the scales. Mean scores for each scale were calculated by dividing the sum of the item scores by the number of items. Thus, scores are on the same scale as the ratings (1–4 for REF and 1–5 for all others). All scales evidenced similar scores to those reported in previous studies, except for CT disposition, for which the mean score was not reported in the original study.

**Table 1:** Descriptive Statistics, Gender Differences, and Internal Consistencies of Study Variables (N = 760).

	Men^a^		Women^b^		Total		Gender Difference	α
	*M*	*SD*	*M*	*SD*	*M*	*SD*	*t*	
Critical Thinking Disposition	3.39	0.50	3.38	0.47	3.39	0.49	0.36	.90
Awareness of logical thinking	3.10	0.61	2.94	0.60	3.02	0.61	3.77***	.86
Inquiry-mindedness	3.63	0.70	3.83	0.64	3.74	0.68	−4.07***	.85
Evidence-based judgment	3.57	0.73	3.37	0.67	3.47	0.71	3.97***	.46
Objectiveness	3.51	0.62	3.56	0.62	3.53	0.62	−1.11	.75
Responsibility to Continue Thinking	3.23	0.70	3.16	0.71	3.19	0.71	1.53	.87
Detached Awareness^c^	2.46	0.53	2.38	0.57	2.42	0.55	2.08*	.72
Worry^d^	3.04	0.76	3.12	0.78	3.08	0.77	−1.49	.92

* *p*<.05;

*** *p*<.001.

a *n* = 360.

b *n* = 400.

c Measured by the Refraining from Catastrophic Thinking Scale.

d Measured by the Penn State Worry Questionnaire.

All internal consistency coefficients (except for Evidence-based judgment, α = .46) exceeded.72, indicating acceptable reliability; five of the eight alpha coefficients obtained exceeded.85, indicating excellent reliability. Although low α for Evidence-based judgment is a concern, this is understandable because it has only three items. In addition, our main analysis focused on total CT disposition according to Hirayama and Kusumi [34], who supported the use of total score based on confirmatory factor analysis (see Methods). As post-hoc subscale-wise analyses were also conducted, however, caution is recommended in interpreting the results involving this subscale.

Although the age and grade (year in university) distribution was highly skewed (skewness was 9.52 and 2.82, respectively), CT disposition total and Awareness of logical thinking were correlated with age (Spearman’s *r* = .14, *p*<.001, for both). Responsibility to continue thinking was positively related to grade (Spearman’s *r* = .11, *p*<.01). In addition, there were gender differences for three out of four CT disposition subscales and detached awareness. Awareness of logical thinking, Evidence-based judgment, and detached awareness were higher for men, while Inquiry-mindedness was higher for women. Controlling for age and grade in the subsequent analysis did not change the overall pattern of the following results; therefore, age and grade were not included in subsequent analyses. In addition, we examined gender invariance of the mediational model.

Table 2 summarizes the correlations among study variables. CT disposition was positively related to both potential mediators (*r* = .24 to responsibility to continue thinking and *r* = .36 to detached awareness; both *p*<.001) and negatively correlated with worry (*r* = −.20; *p*<.001). In addition, responsibility to continue thinking was positively related to worry (*r = *.47; *p*<.001), while detached awareness was negatively related to worry (*r* = −.61; *p*<.001).

**Table 2:** Correlation Among Study Variables (N = 760).

		2	3		4		5	6	7	8
1	Critical Thinking Disposition	.85***		.76***		.53***	.70***	.24***	.36***	−.20***
2	Awareness of logical thinking	1.00		.42***		.40***	.45***	.08*	.38***	−.28***
3	Inquiry-mindedness			1.00		.23***	.38***	.24***	.25***	−.12***
4	Evidence-based judgment					1.00	.38***	.27***	.06	.06
5	Objectiveness						1.00	.21***	.20***	−.07
6	Responsibility to Continue Thinking							1.00	−.19***	.47***
7	Detached Awareness^a^								1.00	−.61***
8	Worry^b^									1.00

* *p*<.05;

*** *p*<.001.

a Measured by the Refraining from Catastrophic Thinking Scale.

b Measured by the Penn State Worry Questionnaire.

### Multiple Mediation Analysis

**Figure 1:**
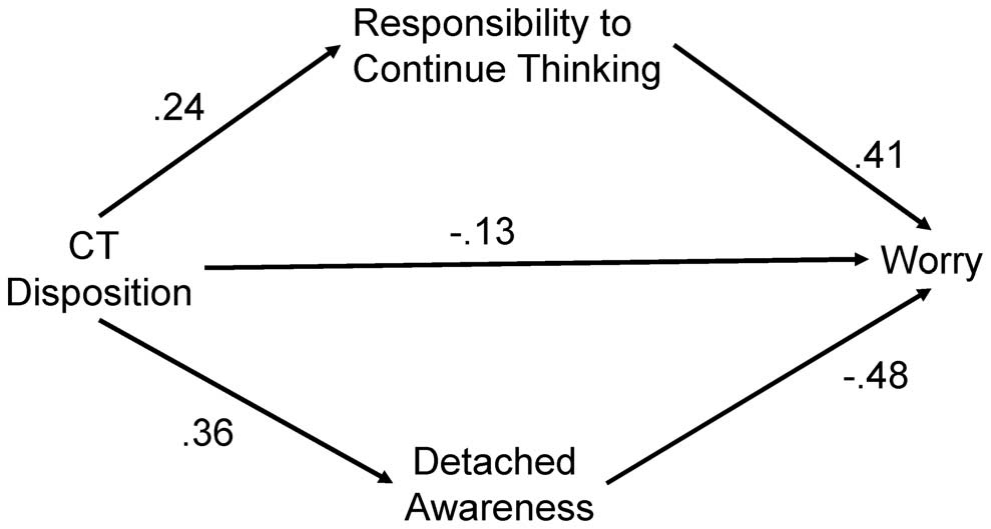
Multiple mediational model predicting worrying by CT disposition (N = 760). The values with one-headed arrows are the standardized regression coefficients (all *p*<.001).

Standardized regression coefficients for the multiple mediation model are depicted in Figure 1. All standardized regression coefficients were statistically significant (*p*<.001), and the model explained 51% of the variance of worrying. The statistical significance of two indirect pathways from CT disposition to worry was examined by bootstrapping estimation [50], [51]. If the 95% confidence interval (CI) of an estimated effect does not include zero, this effect is considered significant at the 5% level. Indirect effects were significant for both responsibility to continue thinking (*B* = .15; *SE* = .03; 95% CI = .10–.22) and refraining from catastrophic thinking (*B* = −.27; *SE* = .03; 95% CI = −.34–−.21). Mallinckrodt et al. also recommended the test of joint significance as a statistical test for mediation, which requires a researcher to indicate that each path is significant [49]. As indicated by the statistical significance of all paths, the results also satisfied the latter criteria. These results indicate that the effect of worry on CT disposition was partially mediated by the two potential mediators, with a significant direct effect as well.

### Invariance of the Mediational Model across Genders

Gender difference in the mediational model was examined. In addition to gender differences in the present data (Table 1), a previous study also found gender differences in some facets of CT disposition [52]. Multiple population analysis was conducted by using AMOS 20.0 to compare models with/without the constraints of invariance of parameters across genders. Specifically, four models with differing strengths of invariance were compared: Model 1 has no invariance constraints; parameters were free to differ between genders. In Model 2, regression weights were set to be equal. Model 3 additionally required covariances to be equal. The strongest, Model 4, further requires residuals to be equal. Akaike information criterion (AIC) and Browne-Cudeck criterion (BCC) are fit indices suitable for model comparison in which smaller values indicate better fit [53]. AICs for Models 1–4 were 40.00, 40.77, 40.01, and 39.45, respectively; the corresponding BCCs were 40.54, 41.17, 40.38, and 39.71, respectively. Therefore, the strongest constraints of invariance (indicating that the models are strictly the same across genders) yielded the best fit, indicating that the mediational model did not differ across genders. Indeed, root mean square error of approximation (RMSEA) for Model 4 was.035, indicating close fit.

### The Effects of CT Disposition Subscales on Worrying

Mediation analyses were repeated with CT disposition subscales as independent variables. The same procedures as used in total score analysis were repeated for each subscale [51], while controlling for the remaining three CT disposition subscales as covariates. Table 3 presents a summary of these mediation results. Indirect effects via responsibility to continue thinking were significant (i.e., 95% CI did not include zero) for all four subscales, while those via detached awareness were significant for three out of the four subscales, except for Objectiveness. A direct effect of CT disposition subscales on worrying was only significant for Awareness of logical thinking. In addition, although the indirect effect via responsibility to continue thinking was positive for total CT disposition scores (Figure 1), that for Awareness of logical thinking was negative. Similarly, while the indirect effect via detached awareness was negative for total CT disposition scores (Figure 1), that for Evidence-based judgment was positive.

**Table 3:** The Effect of Critical Thinking Disposition Subscales on Worrying, Mediated by Responsibility to Continue Thinking and Detached Awareness.

	Indirect effect via Responsibility to Continue Thinking				Indirect effect via Detached Awareness				Direct effect on worrying			
			95% CI				95% CI				95% CI	
Critical Thinking Subscales	PE	*SE*	*LL*	*UL*	PE	*SE*	*LL*	*UL*	PE	*SE*	*UL*	*LL*
Awareness of logical thinking	−.006	.002	−.009	−.002	−.017	.002	−.021	−.013	−.013	.003	−.020	−.007
Inquiry-mindedness	.009	.002	.006	.014	−.006	.002	−.010	−.002	−.006	.003	−.013	.001
Evidence-based judgment	.035	.006	.024	.048	.022	.007	.010	.036	.016	.011	−.005	.037
Objectiveness	.007	.003	.001	.013	−.004	.004	−.011	.003	.002	.005	−.008	.013

*Note:* CI = bias-corrected confidence interval; PE = point estimate; *LL* = lower limit; *UL* = upper limit.

### Alternative Mediational Model

Finally, an alternative mediational model was also examined. We examined the possibility that worry mediates the effects of responsibility to continue thinking and detached awareness on CT disposition. Clinical interventions thought to reduce responsibility to continue thinking and/or enhance detached awareness were found to reduce worry [30], [54]. Therefore, the pathways from responsibility to continue thinking and detached awareness to worrying were not changed. Instead, we reversed the pathways between this triad and CT disposition.

**Figure 2:**
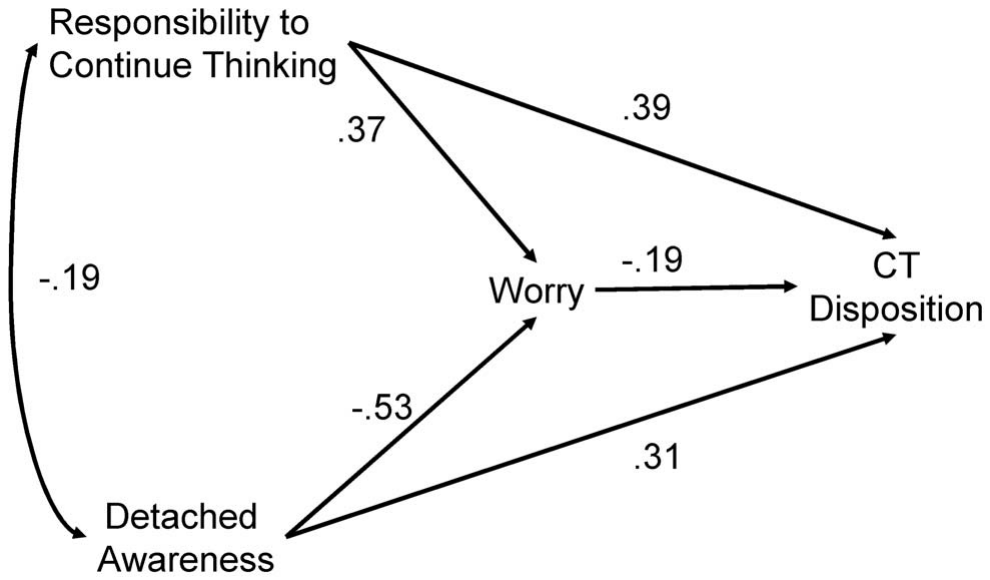
An alternative mediational model predicting CT disposition by responsibility to continue thinking and detached awareness, mediated by worry (N = 760). The values with one-headed arrows are the standardized regression coefficients. The value on the two-headed arrow is a correlation coefficient (all *p*<.001).

Figure 2 depicts the alternative model with standardized regression coefficients (all were significant at *p*<.001). Both responsibility to continue thinking and detached awareness had direct effects on worrying (βs = .37 and −.53, respectively) and CT disposition (βs = .39 and.31, respectively); while worrying negatively predicted CT disposition (β = −.19). Indirect effects on CT disposition through worry were significant for both responsibility to continue thinking (*B* = −.05; *SE* = .01; 95% CI = −.08–−.02) and detached awareness (*B* = .10; *SE* = . 02; 95% CI = .05–.14).

As the original and alternative models cannot be distinguished by model fitness, we examined the variance explained of the final dependent variable. Although Kazdin stated that variance explained per se did not indicate mediation [55], we still believe that it provides clues as to the ordering of a given set of variables in the path diagram. The direct effect of worry on CT disposition (β = −.19) was small compared to other standardized regression weights. Indeed, worry explained only a small unique variance of CT disposition. When entered in the first step in the regression predicting CT disposition, worry explained 4% of the variance, while responsibility to continue thinking and detached awareness explained an additional 20% in the second step. Similarly, when responsibility to continue thinking and detached awareness were entered in the first step, it explained 22% of the variance of CT disposition, while worry explained an additional 2% in the second step. Furthermore, variance of CT disposition explained in the alternative model (24%) was smaller than that of worrying in the original model in Figure 1 (51%).

## Discussion

CT disposition enhanced worrying by enhancing responsibility to continue thinking, while reduced worrying by enhancing the detached awareness of negative thoughts (Figure 1). CT disposition also demonstrated a direct negative relationship to worry. These relationships were consistent across genders. Second, CT disposition subscales differed in their relative contributions to the positive/negative effects of CT disposition on worrying. Finally, an alternative path model in which worry mediated the effect of responsibility to continue thinking and detached awareness on CT disposition was examined; this did not perform as well as the original model in Figure 1.

One of the chief findings of this study was that CT disposition enhanced worrying by promoting the responsibility to continue thinking. The positive relationship between CT disposition and the responsibility to continue thinking, and in turn to worrying, means that we should be cautious of the negative concomitant efforts of CT. Motivation to use CT, reflected in CT disposition, may lead to a persistence conducive to worry. This finding is also consistent with our everyday experience that deliberate thinking is quite effortful. Furthermore, there is evidence that effortful cognitive processing (e.g., a working memory task) is related to increased heart rate [56], [57] and reduced heart rate variability [57], the latter being correlated with worrying [58]. In addition, Pomerantz, Saxon, and Oishi found that goal investment and perceived effort toward goals are related to worrying and positive emotions [59]. Although rigid and inflexible thinking, as represented by responsibility to continue thinking, is not technically CT, such a correlation indicates that people are likely to become perseverant when they voluntarily adopt CT.

Although increasing awareness of the positive effects of CT may motivate more people to engage in it, failure to raise awareness of its accompanying distress may discourage the voluntary use of CT. When facilitating CT, it is important to mention that people may experience worry when engaging in it. One study found that acknowledging the feelings associated with a task (informing participants that their feelings are understandable and acceptable) enhanced participants’ motivation to continue to engage in the task [60].

One encouraging finding is that CT disposition itself had anti-worry effects. Previous studies found that when responsibility to continue thinking was controlled for, a negative pathway from problem-solving to worrying emerged that was not evident in simple correlations [17], [23]. This study enhanced our understanding by showing that this negative relationship was partially mediated by detached awareness, suggesting that CT works similarly to CBT in reducing worrying by decoupling negative thoughts and later processing. Although the direct effect of CT disposition on worrying was also significant, indirect effect through detached awareness was 1.37 times larger than direct effect (95% CI = .82 to 2.88). Recent evidence suggests that “decoupling” is an effective strategy for maintaining motivation in the face of pain, even without reduction in pain itself [61]. In a study conducted by Páez-Blarrina et al., electric shocks were given occasionally during a task [61]. Participants were required to determine whether to continue regardless of the pain or to quit so as to avoid the painful shocks. Those who were told a story about people working with pain in order to feed their families persisted longer than those who were told about people who stopped studying because of pain. Therefore, persistence was achieved not by reduced pain (participants in both group experienced the same intensity of pain), but by the disconnection of pain from subsequent behavior. The results of this experiment suggest that people can be motivated to use CT, even when it poses some distress (increased worry). While this study revealed that CT disposition could reduce worrying, whether and/or how CT can enhance the decoupling of worrying and the discontinuation of CT are interesting questions for future research.

Subscale-level mediation analyses provided clues for further interpreting the double-sided effect of CT disposition on worrying. First, a direct effect on worrying was observed only for one out of four subscales, Awareness of logical thinking, again suggesting that the effect of CT disposition was largely mediated by two mediators. Second, the subscales differed from each other in their relative contribution to CT disposition’s positive/negative relationship to worrying. Awareness of logical thinking reduced worrying via both responsibility to continue thinking and detached awareness. Therefore, this dimension was exclusively related to reduced worry. As this subscale includes items reflecting confidence in using CT (e.g., “I have confidence in thinking accurately.”), the result is consistent with a link between worry and low problem-solving confidence [17], [20], [23]. Furthermore, the fact that a direct effect on worry was found only for Awareness of logical thinking implies that the direct negative effect of CT disposition on worrying in Figure 1 may be largely driven by confidence in using CT. Conversely, Evidence-based judgment was positively related to worrying via both responsibility to continue thinking and detached awareness, suggesting that this dimension is exclusively related to increased worry. Thus, a strong need for evidence may enhance worrying. This is consistent with a study that indicated that worry is associated with elevated evidence requirements [62]. However, replication is needed for the results involving Evidence-based judgment because of its low internal consistency. Finally, Inquiry-mindedness was positively related to both responsibility to continue thinking and detached awareness, mirroring the double-sided effects observed with the total CT disposition scores, but without a direct effect on worrying. Actively open-minded thinking, a scale with items similar to Inquiry-mindedness, was found to predict both more information-seeking in decision making [63] and evaluation of arguments independent of one’s prior beliefs [64]. Increased information-seeking may also lead to persistence as reflected by the responsibility to continue thinking, while thinking independent of prior beliefs may enhance detached awareness of negative thoughts. Although more information-seeking led to correct decisions in their experiment, Haran et al. admitted that excess searches for information could be counter-productive [63].

Examination of an alternative model suggested that the present finding (that CT disposition had double-sided effects on worrying partially mediated by the responsibility to continue thinking and detached awareness) may be more plausible than worry mediating the effect of these two variables on CT disposition, because in the alternative model, the effect of worry (mediator) on CT disposition (dependent variable) was not strong. In fact, responsibility to continue thinking and detached awareness explained much more of the variance of CT disposition than worrying. Furthermore, responsibility to continue thinking and detached awareness explained much more of the variance of worrying in the original model (Figure 1) as well. In the original model (Figure 1), CT disposition explained 4% of the variance when entered in the first step in the regression predicting worry, while responsibility to continue thinking and detached awareness explained an additional 47% in the second step. Similarly, when responsibility to continue thinking and detached awareness were entered in the first step, they explained 50% of the variance of worry, while CT disposition explained an additional 1% in the second step. This suggests that responsibility to continue thinking and detached awareness are more suitable as mediators rather than independent variables. Clinical interventions thought to reduce responsibility to continue thinking and/or enhance detached awareness were found to reduce worry [30], [54]; thus, worry may not be an independent variable. Therefore, worry may be better as a dependent variable rather than as a mediator. In fact, the variance of worrying in Figure 1 was explained to a larger degree than that of CT disposition in Figure 2.

Finally, limitations and future directions should be discussed. First, because indirect effects via worrying were statistically significant, an alternative model cannot be rejected. Thus, future longitudinal study will determine causality or elucidate the reciprocal influences, if any. Second, it would be useful to examine other variables that could explain the current relationships. The actual execution of CT may determine its effect on the current mediators or dependent variable. In addition, as noted in the Introduction, Chan et al. found that belief in certainty of knowledge predicted unsuccessful execution of CT beyond cognitive ability [6]. Indeed, worriers have been found to be intolerant of uncertainty [65]. Finally, analogous to studies that predicted performance on reasoning tasks by cognitive styles [7], [8], future study might examine the effect of CT disposition and two mediators on performance-based tasks purported to engage characteristics of worrying, for example, a catastrophizing task [44] or thought sampling while focusing on breathing [66]. When researchers measure both the frequency of worrying and the negative reaction to it during a given period, a low correlation between them may index detached awareness of negative thinking [67]. HR during or after stressful cognitive tasks may also be used [47], [48]. Cognitive-style studies also included cognitive ability as one of the predictors [7], [8]. In the present context, executive function may be a candidate cognitive ability to reduce worrying [68].

In conclusion, although CT disposition has many positive effects, the multiple mediation analyses conducted in this study revealed that the relationship between CT disposition and worrying was mediated by two constructs: one through responsibility to continue thinking and the other via detached awareness of negative thinking. There was also a direct effect from CT disposition, possibly driven by confidence in using CT. Although the pathway via responsibility to continue thinking is consistent with our everyday experience of deliberate thinking as hard work, this also suggests that we should be aware of this effect, lest it discourage people from using CT. The other pathway, through which CT disposition reduced worrying via detached awareness, is promising, and further suggests that the decoupling of worrying and the later use of CT is an interesting topic for future research.

## Supporting Information

Appendix S1 (DOC)
