# Supplementary material for: The Dual Effects of Critical Thinking Disposition on Worry
Source: PLoS One. 2013 Nov 20;8(11):e79714. doi: 10.1371/journal.pone.0079714 (PMC3835852; doi:10.1371/journal.pone.0079714)
Supplement: Appendix S1 — (DOC) [file pone.0079714.s001.doc]

**Appendix**

###### Sample items

##### Critical Thinking Disposition^a^

I have confidence in thinking accurately (Awareness of logical thinking).

I am good at thinking systematically about complex problems (Awareness of logical thinking).

I like to learn as much stuff as possible, irrespective of its immediate utility (Inquiry-mindedness).

I enjoy having discussions with people whose opinions are different from mine (Inquiry-mindedness).

I don't believe in anything without a bit of skepticism (Evidence-based judgment).

I examine as much evidence as possible when making judgments (Evidence-based judgment).

When making a decision, I try to be objective (Objectiveness).

I try to consider things from as many angles as possible, rather than from a few limited perspectives (Objectiveness).

##### Responsibility to Continue Thinking

I should continue thinking until I find out better solutions.

It is irresponsible to stop thinking.

I think I should keep thinking until I find a better solution.

I have to keep thinking about this problem over and over.

I have to collect more information about this problem.

##### Detached Awareness^b^

Even though I do not feel good, I don't think catastrophically.

Even if the bad consequences of the problem come to mind, I can reassure myself that they are nothing more than my imagination.

Even in such a situation, I keep bright hope and think that I can change adversity into benefit.

I don't develop a negative scenario from the situation.

##### When I start thinking about the situation seriously, I can stop it for a while.

##### Penn State Worry Questionnaire

If I don't have enough time to do everything, I don't worry about it.^c^

My worries overwhelm me.

I don't tend to worry about things.^c^

Many situations make me worry.

I know I shouldn't worry about things, but I just can't help it.

^a^ Names of the four subscales in parentheses.

^b^ Measured by the Refraining from Catastrophic Thinking Scale.

^c^ Reversed-score items (higher scores indicate lower worrying).
